# An Unusual Cause of Secondary ITP in a 34-Year-Old Hispanic Male

**DOI:** 10.1155/2021/7965930

**Published:** 2021-12-23

**Authors:** Ayrton Bangolo, Mohamed Ahmed, Ali Atoot, Ashraf Mahmoud, Chibuzo Agbakwuru-Onyike, Maria Karina Larni Y. Palad, Michael Sciarra, Adam Atoot

**Affiliations:** ^1^Department of Internal Medicine, Hackensack Meridian Health Palisades Medical Center, North Bergen, NJ, USA; ^2^Department of Anesthesia, Hackensack University Medical Center, Hackensack, NJ, USA; ^3^Department of Family Medicine, Hackensack Meridian Health Palisades Medical Center, North Bergen, NJ, USA; ^4^University of the East Ramon Magsaysay, Quezon, Philippines; ^5^Department of Gastroenterology, Hackensack Meridian Health Palisades Medical Center, North Bergen, NJ, USA

## Abstract

Secondary immune thrombocytopenic purpura (ITP) associated with *Helicobacter pylori* (*H. pylori*) infection has been described in the literature. It appears to have a geographic distribution; mostly encountered in countries with a higher prevalence for *H. pylori* such as Italy or Japan. *H. pylori* eradication has been recommended in the management of ITP with studies showing improvement in the platelet count in some patients. Substantial platelet count increases in patients with severe thrombocytopenia (platelet counts <30 × 10^3^ microliter), however, are uncommon with *H. pylori* treatment alone. Here, we present a 34-year-old Hispanic male with worsening chronic thrombocytopenia that resolved following eradication of his *H. pylori* infection. Herein, we highlight a rare and reversible cause of secondary ITP. With this case report, we hope to encourage physicians to include *H. pylori* testing in the evaluation of thrombocytopenia.

## 1. Introduction

Immune thrombocytopenic purpura (ITP) is an acquired disorder in which there is immune-mediated destruction of platelets with possible inhibition of platelet release from the megakaryocytes [1]. In adults, it is a more chronic disease; however, some will experience spontaneous remission which will usually occur within months of the initial diagnosis. ITP is considered secondary if it is associated with an underlying disorder [1]. Several studies have recently shown improvement in platelet counts after eradicating *Helicobacter pylori* (*H. pylori*) infection which suggests an association between the two conditions [2]. It appears that response rates are higher in countries where *H. pylori* infection is endemic and in patients with milder degrees of thrombocytopenia [3, 4]. Herein, we present a 34-year-old male with ITP and worsening platelet levels who was found to have *H. pylori* infection. Following treatment of his infection, the patient had significant improvement and normalization of his platelet count. With this case report, we aim to encourage clinicians to include *H. pylori* in the differential diagnosis of ITP.

## 2. Case Presentation

This is a 34-year-old Hispanic male with no significant past medical history who presented to the clinic for evaluation of a gnawing, moderate, intermittent epigastric pain that started 3-4 months prior to the visit. The pain improves with meals and is associated with reflux and early satiety. Notably, the patient was told he had “low platelets” on his annual physical the year prior to our encounter. He denied any diarrhea, nausea, vomiting, spontaneous bruising, epistaxis, or gingival bleeding. He also denied any intravenous drug use or any dangerous sexual behaviors. He has never been hospitalized and did not undergo any surgical procedures in the past. The family history was noncontributory.

Physical examination was significant for epigastric tenderness on palpation but was otherwise unremarkable. Laboratory results revealed a positive urea breath test as well as worsening thrombocytopenia with a platelet count of 50 × 10^3^ microliter (uL) (150–450 × 10^3^) from a platelet count of 91 × 10^3^ uL the year prior as shown in Table 1. The remainder of the laboratory evaluation, including a complete blood count, lipid profile, and urinalysis was unremarkable. The abdominal ultrasound was unremarkable except for a trace effusion between the liver and diaphragm. Further evaluation with a noncontrast computed tomography (CT) of the abdomen/pelvis showed no abdominal pathology.

The patient was started on a 10-day regimen triple therapy (amoxicillin, clarithromycin, and lansoprazole) for eradication of *Helicobacter pylori* (*H. pylori*). One week after initiation of therapy, repeat laboratory results revealed significantly improved platelet levels, 105 × 10^3^ uL. Additional workup for thrombocytopenia was ordered including a hepatitis panel, human immunodeficiency virus testing, heparin-induced thrombocytopenia antibodies, and a serotonin release assay which were all within normal limits.

The patient was reevaluated 5 weeks after completion of the triple therapy. He reported complete resolution of his abdominal pain. The urea breath test was negative as well as the stool antigen for *H. pylori*. The laboratory results revealed continuous improvement and normalization of the platelet levels, 178 × 10^3^ uL.

## 3. Discussion

Immune thrombocytopenia (ITP, also called idiopathic thrombocytopenic purpura or immune thrombocytopenic purpura) is an acquired thrombocytopenia caused by autoantibodies against platelet antigens. It is one of the more common causes of thrombocytopenia in otherwise asymptomatic adults [1]. ITP is termed secondary if it is associated with an underlying disorder; autoimmune disorders, particularly systemic lupus erythematosus (SLE), and infections, such as human immunodeficiency virus (HIV) infections and hepatitis C, are also common causes. The association of ITP with *Helicobacter pylori* (*H. pylori*) infection is unclear [1]. Some studies show that eradication of *H. pylori* with antibiotics result in marked platelet count increases in patients with ITP [3]. Patients with acute *H. pylori* are often asymptomatic or develop mild self-limited dyspeptic symptoms and may not seek medical attention and thus are not immediately investigated [5]. Our patient had a one-year history of acquired unexplained thrombocytopenia and developed dyspeptic symptoms overtime. He was not initially worked up for *H. pylori* a year prior to our encounter, given the lack of dyspeptic symptoms at the time. He did not endorse any signs or symptoms related to thrombocytopenia. The patient also denied any personal or family history of bleeding disorders or autoimmune conditions.

ITP is a diagnosis of exclusion which is made in patients with isolated thrombocytopenia. The diagnosis of ITP requires that other potential causes of thrombocytopenia, such as recent infections, medications, rheumatologic disorders, or liver disease, be excluded [6]. Several guidelines recommend obtaining a peripheral blood smear to confirm that thrombocytopenia is not artifactual due to platelet clumping and HIV and hepatitis C testing [6–8]. Testing for *H. pylori* is appropriate in patients with gastrointestinal symptoms suggestive of infection because of a reported association between ITP and *H. pylori* infection. Routine screening for *H. pylori* may be reasonable in asymptomatic individuals as well [4]. Antiplatelet antibody testing has low sensitivity and is not recommended [9]. Our patient was not taking any medications including any herbal supplementation to explain his findings. He had no history of rheumatologic disorders, and the liver function test was unremarkable. A year prior to our encounter, the patient had unexplained thrombocytopenia but did not have any gastrointestinal symptoms. It was his dyspepsia and early satiety a year later that prompted evaluation for *H. pylori* infection. However, even despite the lack of dyspeptic symptoms, the patient should have been evaluated for *H. pylori* as part of the workup of unexplained thrombocytopenia.

The treatment of ITP uses drugs that decrease reticuloendothelial uptake of the antibody-bound platelet, decrease antibody production, and/or increase platelet production. Patients with platelet counts >30 × 10^3^ *μ*L appear not to have increased mortality related to the thrombocytopenia and usually do not require treatment with intravenous immune globulin (IVIG) or corticosteroids [1]. IVIG or corticosteroids are usually reserved for bleeding, elective surgery, or severe thrombocytopenia (<30 × 10^3^ microliter) without bleeding [10]. A systematic review of the English literature concluded that, in secondary ITP, there is improvement of thrombocytopenia with *H. pylori* eradication [4]. Our patient had a platelet level above 30 × 10^3^ uL, was not bleeding, and was not scheduled for any surgeries. Thus, he was not treated with corticosteroids or IVIG. He was found to have a positive urea breath test and was initiated on triple therapy for *H. pylori* eradication. Following *H. pylori* eradication, the platelet levels normalized, an outcome that is consistent with findings in the literature.

## 4. Conclusion

Immune thrombocytopenic purpura (ITP) is an acquired disorder that can be associated with *Helicobacter pylori* (*H. pylori*) as previously reported in the literature. *H. pylori* eradication has been associated with improvement and normalization of the platelet levels, especially in patients with nonsevere thrombocytopenia. With this case report, we hope to contribute to the growing literature of *H. pylori* associated with ITP. This case report has the objective to encourage clinicians to include *H. pylori* in the workup of unexplained thrombocytopenia, even without gastrointestinal symptoms.

## Figures and Tables

**Table 1:** Laboratory values.

	Laboratory values
White blood cells	5.5 × 10^∗^3 (3.4–10.8 × 10^∗^3) microliter (uL)
Red blood cells	5.0 × 10^∗^6 (4.14–5.80 × 10^∗^6) uL
Platelet count on admission	50 × 10^∗^3 (150–450 × 10^∗^3) uL
Platelet count a year ago	91 × 10^∗^3 (150–450 × 10^∗^3) uL
Platelet count 1 week into therapy	105 × 10^∗^3 (150–450 × 10^∗^3) uL
Platelet count 5 weeks after triple therapy	178 × 10^∗^3 (150–450 × 10^∗^3) uL
Erythrocyte sedimentation rate	3 (0–15) millimeter per hour (mm/hr)
C-reactive protein	<1 (0–10) milligram per liter (mg/L)
Hepatitis B surface antigen	Negative
Hepatitis B surface antibody, quantitative	>10,000 (immunity >9.9) milli-international units per milliliter (mIU/mL)
Aspartate aminotransferase	19 (0–40) international units per liter (IU/L)
Alanine transaminase	25 (0–44) IU/L
Human immunodeficiency virus antigen/antibody 4th generation	Nonreactive
Hepatitis C antibody	<0.1 (0.0–0.9) signal-to-cutoff ratio (s/co ro)
Antinuclear antibody	Negative
Serotonin release assay	Negative

## Data Availability

All data generated or analyzed during this study are available from the corresponding author upon request.
